# Cardiovascular Risk Evaluation in a Latin American Population With Severe Mental Illness: An Observational Study

**DOI:** 10.62641/aep.v53i4.1904

**Published:** 2025-08-05

**Authors:** Juan Rivas, Carlos Miranda, Anita Restrepo, Mauricio Hernández, Jose Miguel Erazo, María Juliana Martínez, Jennifer Lasso, Laura López, Juan Esteban Gómez-Mesa

**Affiliations:** ^1^Hospital Departamental Psiquiátrico Universitario del Valle, 76003 Cali, Colombia; ^2^Departamento de Psquiatría, Universidad del Valle, 76003 Cali, Colombia; ^3^Departamento de Psquiatría, Fundación Valle del Lili, 76003 Cali, Colombia; ^4^Facultad de Ciencias de la Salud, Universidad Icesi, 76003 Cali, Colombia; ^5^Department of Psychology, University of Chicago, Chicago, IL 60627, USA; ^6^Departamento de Cardiología, Fundación Valle del Lili, 76003 Cali, Colombia

**Keywords:** severe mental illness, cardiovascular risk factor, Framingham calculator, schizophrenia, bipolar disorder, psychotic depression

## Abstract

**Background:**

Patients with severe mental illness (SMI) have a life expectancy that is 15 to 20 years shorter than that of the general population primarily due to cardiovascular disease (CVD), which is a leading cause of mortality. Contributing factors include unhealthy lifestyles, physical inactivity, diet, smoking, specific medications, and obesity. This study seeks to describe risk factors and cardiovascular risks (CVR) among hospitalized patients with SMI at a specialized mental healthcare center.

**Methods:**

This retrospective study analyzed demographic and clinical data from hospitalized patients between January and December 2022. The Framingham 10- and 30-year risk scores were used to assess CVR, and logistic regression was employed for statistical analysis to compare results and determine significant differences.

**Results:**

The study consisted of 366 patients, of whom 47.2% were women, aged 18 to 81. Women were, on average, older than men, with mean ages of 44.7 and 37.8 years, respectively (*p* < 0.001). Additional demographic characteristics show that 54.9% had finished high school and 81.1% were single. The biggest CVR factors in our sample were female sex, higher academic level, systolic blood pressure above 150 mmHg, triglyceride levels above 150 mg/dL, fasting glucose values above 100 mg/dL, cigarette smoking, and a family history of CVD.

**Conclusions:**

The research shows an incidence of CVD risk among patients with SMI. Multiple lifestyle and medical factors correlate with an increased mortality risk over 10 and 30 years. These findings highlight the need to control modifiable risk factors such as blood pressure (BP), serum lipids, glucose levels, smoking habits, and possible medication side effects to positively impact survival and quality of life in this group.

## Introduction

Severe mental illnesses (SMIs) are characterized by the presence of psychotic symptoms, and include schizophrenia (SCHZ), bipolar disorder (BD), and psychotic depression (PD) [1]. In 2019, the global prevalence of SCHZ, BD, and major depressive disorder was 23.6, 39.5, and 279.6 million people, respectively [2]. In the United States, 5–10% of adults have been diagnosed with SMI [3]. In Colombia, the estimated prevalence is 0.3–1.6% for SCHZ, 1–2% for BD, and 4.2–20.8% for depression [4, 5].

Patients with SMI have a life expectancy 15 to 20 years shorter than that of the general population [6, 7]. The relative risk of death from all causes affecting these patients is 2.22 compared to the general population [8]. Mortality in patients with SMI is associated with cardiovascular disease (CVD), cancer, chronic obstructive pulmonary disease, and renal disease in 67.3% of cases. Suicide, accidents, and homicides account for 17.5% of deaths, while the remaining causes are undetermined [8, 9].

CVD accounts for 33% of deaths in the general population worldwide [10]. In Colombia, it was reported that 29.2% of deaths are associated with CVD [11]. In patients with SMI, CVD is the leading cause of mortality [9, 12]. Mortality rates in BD and SCHZ range from 25% to 50% [9, 13, 14]. The most common CVDs across these patients include ischemic heart disease, non-ischemic heart disease, cerebrovascular diseases, and other circulatory diseases [9]. In the United Kingdom, mortality rates differ by sex in patients with SMI, with rates ranging from 23.9% in men to 17.6% in women [15]. Therefore, it becomes important to assess cardiovascular risks (CVR) in these patients [16].

Several factors increase CVR in patients with SMI. Unhealthy lifestyles, lack of exercise, diet, smoking, and obesity have been described as contributing factors [17, 18]. Comparative studies have shown that patients with SMI engage in less physical activity per day compared to the general population [17]. Patients with SCHZ also show higher rates of smoking (68% vs. 35%), diabetes (13% vs. 3%), and hypertension (27% vs. 17%) when compared to the general population [19].

Antipsychotic medication use has been associated with an increase in CVR. Some side effects can lead to weight gain, hyperglycemia, and hyperlipidemia [20, 21]. Additionally, a study suggests a genetic predisposition to CVD in patients with SMI [22].

To our knowledge, there are few studies measuring CVR factors in the Latin American population with SMI. This study aims to describe risk factors and calculate CVR in a population of hospitalized patients with SMI at a specialized mental health center, and compare results between different diagnostic categories.

## Methods

In this retrospective study, demographic and clinical information was collected from patient medical charts to assess CVR profiles in a sample of hospitalized patients diagnosed with SMI. The sample was drawn from Hospital Departamental Psiquiátrico Universitario del Valle in Cali, Colombia, between January 2022 and December 2022. This study was conducted in accordance with the principles set forth in the Declaration of Helsinki by the World Medical Association, and all procedures were approved by the relevant institutional ethics committee. Since this is a retrospective study that does not involve direct patient intervention or the collection of personally identifiable data. However, confidentiality and privacy of the collected information were maintained.

### Demographic and Psychiatric Information

Demographic information obtained included sex, age, highest academic level achieved, and marital status. Psychiatric diagnoses were based on the International Statistical Classification of Diseases and Related Health Problems 10th Revision (ICD-10) diagnostic criteria and, for this analysis, they were grouped into three diagnostic categories or spectrums based on Diagnostic and Statistical Manual of Mental Disorders, Fifth Edition (DSM-V) and ICD-10: (1) “SCHZ spectrum”, which included SCHZ and Schizoaffective Disorders; (2) “affective disorders spectrum”, which included Bipolar and Depressive Disorders; and (3) “other disorders spectrum”, which included organic mental disorders. Supplementary files included a comprehensive list of diagnostic codes in the sample and their respective categorization (Appendix Table 5). Patients also reported the number of years since their psychiatric diagnosis, antipsychotic medication prescribed, if any, current dosage, and whether they had a family history of psychiatric illness.

### Inclusion and Exclusion Criteria

Male and female patients over 18 years of age diagnosed with severe mental illness and hospitalized between 1 January and 31 December 2022 were included. Patients with incomplete demographic or clinical data were excluded.

### CVR Factors

Medical charts contained information on a variety of cardiovascular variables and comorbidities, including a history of hypertension, diabetes, and other heart diseases. Body Mass Indices (BMIs) were calculated from available weight and height data. Measures also included systolic and diastolic blood pressure (BP), triglyceride levels, cholesterol levels (total, high-density lipoprotein [HDL], and low-density lipoprotein [LDL]), fasting glucose, and current and past cigarette use. The expected reference values of lipid profile are as follows:

• Triglycerides: Normal: <150 mg/dL, borderline high: 150–199 mg/dL and high: ≥200 mg/dL;

• Total Cholesterol: Desirable: <200 mg/dL, borderline high: 200–239 mg/dL and high: ≥240 mg/dL;

• LDL: Optimal: <100 mg/dL, near optimal/above optimal: 100–129 mg/dL, borderline high: 130–159 mg/dL, high: 160–189 mg/dL, and very high: ≥190 mg/dL;

• HDL: Low: <40 mg/dL (men) and <50 mg/dL (women), acceptable: 40–59 mg/dL, and high (protective against heart disease): ≥60 mg/dL.

Ten-year Framingham Risk Scores were calculated using the following formula [23]:

RiskFactors = (ln(Age) × 3.06117) + (ln(Cholesteroltot) × 1.12370) – (ln(CholesterolHDL) × 0.9326) + (ln(BPsystolic) × FactorSistólica) + CigaretteUse + Diabetes – 23.9802

RiskScore = 100 × (1 – 0.88936^eRiskFactors^)

According to the results and based on the Framingham CVR classification, the individuals were categorized as low (≤9.99), moderate (10–19.9), high (20–29.9) and very high (≥30) CVR.

Thirty-year Framingham risk scores were calculated based on the risk calculator created by Pencina *et al*. [24], which includes total and HDL cholesterol values instead of BMI values as inputs. In the 30-year Framingham risk score, individuals were categorized as low (≤9.99), moderate (10–19.9), high (20–29.9), and very high (≥30) CVR.

### Statistical Analysis

Given the number of records available, the method used to assess normality was the Kolmogorov-Smirnov test. We vectorized the calculation methods and generated simple functions to be implemented in R (R version 4.3.2 (2023-10-31 ucrt) –“Eye Holes”; Copyright © 2023. The R Foundation for Statistical Computing, Vienna, Austria). Sex differences in risk factor levels were investigated using separate logistic regression models for the binary risk variables (systolic BP, diastolic BP, triglycerides, cholesterol total, HDL, and LDL), and fasting glucose and cumulative link models for the ordinal risk variables (BMI, cigarette use, and Framingham 10-Year Risk) using the “ordinal” package [25] in R [26]. See the **Supplementary Materials** for further individual model information.

For CVR comparison using Framingham calculators for risks at 10- and 30-year outcomes, we sorted patients into low, moderate, high, and very high risk categories based on their Framingham scores. Chi-square tests were used to assess differences in distributions of risk categories, whereas the Kruskal-Wallis test was applied to compare continuous variables such as age and BMI. A significant level of 0.05 was established.

The CVR variable in the 30-year Framingham score was dichotomized into two categories to simplify its interpretation and analysis. The variable values were divided as follows:

• 0 = Low Risk: For individuals with a CVR score below 10, indicating low CVR.

• 1 = More than Low Risk: For individuals with a CVR score ranging from 10 to 100, indicating a risk above low CVR.

This dichotomization helps sort participants into two main groups for a clearer analysis of the variables associated with cardiovascular risk over time. A binomial logistic regression model was then performed to assess the influence of each variable on CVR. Statistical analysis was conducted by using the 4.3.1 R version.

## Results

We included 366 patients, of which 47.2% were women. There was no significant difference in the distribution of men vs. women in the sample ($\chi^{2}$ = 1.09, *p* = 0.3). Patient ages ranged from 18 to 81, but women were older than men (44.7 vs. 37.8 years, *p* < 0.001). 54.9% had earned a high school degree and 81.1% were single. As for diagnoses, the percentages were: 52.2% in the SCHZ spectrum, 33.9% in the affective disorders spectrum, and 13.9% in the other disorders spectrum. 54.9% of the population had a mental disease history of more than 10 years. Most patients (76.8%), had no family history of psychiatric illness, whereas 91.8% had no family history of CVD (Table 1).

**Table 1.** **Demographic characteristics**.

Variable		N = 366	%
Sex			
	Female	173	47.2
	Male	193	52.7
Age		41 (Mean)	SD = 14.4
Education			
	No schooling	25	6.8
	Primary	94	25.7
	High school	201	54.9
	Technical and university studies	46	12.6
Marital status			
	Single	297	81.1
	Married	69	18.9
Diagnosis group			
	Schizophrenia spectrum	191	52.2
	Affective disorders spectrum	124	33.9
	Other disorders spectrum	51	13.9
Time since diagnosis			
	<1 year	17	4.6
	1–5 years	86	23.5
	5–10 years	62	16.9
	10–15 years	71	19.4
	>20 years	130	35.5
Family history of psychiatric illness			
	No	281	76.8
	Yes	85	23.2
Family history of cardiovascular disease			
	No	336	91.8
	Yes	30	8.2

SD, standard deviation.

Comparing sociodemographic variables across diagnostic categories, more than half of the sample had finished high school (*p* = 0.019). A higher incidence of diabetes was observed in the SCHZ spectrum (*p* = 0.023), whereas those in the affective spectrum had higher BMI (*p* = 0.002) and a higher prevalence of family history of CVD (*p* = 0.046) (Table 2).

**Table 2.** **Sociodemographic and clinical variables according to psychiatric spectrums**.

Variable	Category	Schizophrenic spectrum		Affective spectrum		Other disorders spectrum		Chi-square	df	*p*-value
		n = 191	%	n = 124	%	n = 51	%			
Sex	Male	118	62.0	37	30.0	38	75.0	42.051	2	0.0001*
	Female	73	38.0	87	70.0	13	25.0			
Marital status	Single	168	88.0	101	82.0	45	88.0	2.902	2	0.234
	Married	23	12.0	23	19.0	6	12.0			
Education	No schooling	14	7.0	3	2.0	8	16.0	15.133	6	0.019*
	Primary	56	29.0	29	23.0	9	18.0			
	High school	102	53.0	71	57.0	28	55.0			
	Technical and university	19	10.0	21	17.0	6	12.0			
History of hypertension	No	160	84.0	109	88.0	47	92.0	2.790	2	0.248
	Yes	31	16.0	15	12.0	4	8.0			
History of diabetes	No	168	88.0	120	97.0	47	92.0	7.568	2	0.023*
	Yes	23	12.0	4	3.0	4	8.0			
Family history - psychiatric illness	No	144	75.0	94	76.0	43	84.0	1.895	2	0.388
	Yes	47	25.0	30	24.0	8	16.0			
Family history - cardiovascular disease	No	175	92.0	110	89.0	51	100	6.139	2	0.046*
	Yes	16	8.0	14	11.0	0	0.0			
Systolic BP (mmHg)	Low <140	153	80.0	86	69.0	40	78.0	4.954	2	0.084
	High >140	38	20.0	38	31.0	11	22.0			
Diastolic BP (mmHg)	Low <90	144	75.0	96	77.0	40	78.0	0.294	2	0.863
	High >90	47	25.0	28	23.0	11	22.0			
Triglycerides** mg/dL	Normal <150	151	79.0	100	81.0	42	82.0	0.315	2	0.854
	High >150	40	21.0	24	19.0	9	18.0			
Total cholesterol** mg/dL	Normal <200	166	87.0	101	82.0	44	86.0	1.834	2	0.400
	High >200	25	13.0	23	19.0	7	14.0			
LDL cholesterol** mg/dL	Normal <100	113	59.0	63	51.0	31	61.0	2.567	2	0.277
	High >100	78	41.0	61	49.0	20	39.0			
HDL cholesterol*** mg/dL	Normal >60	17	9.0	15	12.0	4	8.0	1.131	2	0.568
	Low <60	174	91.0	109	88.0	47	92.0			
Fasting Glucose mg/dL	Low <100	150	79.0	99	80.0	45	88.0	2.426	2	0.297
	High >100	41	22.0	25	20.0	6	12.0			
Cigarette use	Non-smoker	109	57.0	74	60.0	29	57.0	0.237	2	0.888
	Smoker	82	43.0	50	40.0	22	43.0			
BMI classification	Low (<18.5)	12	7.0	4	3.0	4	9.0	20.500	6	0.002*
	Normal (18.5–25)	84	46.0	43	37.0	32	70.0			
	Overweight (25–30)	50	27.0	42	36.0	4	9.0			
	Obese (>30)	38	21.0	28	24.0	6	13.0			

df, degrees of freedom. Chi-Square Test. BMI, Body Mass Index; BP, blood pressure; HDL, high-density lipoprotein; LDL, low-density lipoprotein; dL, deciliters; mg, milligrams; mmHg, millimeters of mercury. *Statistically significant. **The classification of triglycerides, total cholesterol, and LDL is based on the desired values in adults categorized as normal or high. *** The classification of HDL is based on the desired values in adults categorized as normal or low.

A 25 BMI was observed in 45.9% of patients. The proportion of patients with BMI >25 differed by diagnostic spectrum. 60.0% of those with affective disorders, 48.0% with SCHZ spectrum, and 22.0% with other diagnoses had BMIs above this threshold (*p* = 0.002). A family history of CVD was present in 8.0% of patients, all of whom had either SCHZ spectrum or affective disorder diagnoses. 25.0% of patients with SCHZ spectrum and 24.0% with affective disorders reported a family history of psychiatric conditions (Table 2).

When dichotomizing the risk scores into low risk (<10) and more than low risk (10–100), more men were classified in the low-risk category at both 10 and 30 years (*p* = 0.005 for 10 years and *p* = 0.043 for 30 years). Women had a higher proportion in the more than low-risk category at both timeframes. Additionally, it was more prevalent among male smokers than female smokers (56.5% vs. 26.0%, *p* = 0.0001). Regarding BMI, more men were classified as “normal” (54.6% vs. 36.0%, *p* = 0.0001) while more women were classified as “overweight” (59.8% vs. 38.3%, *p* = 0.0001).

No significant sex differences were observed in family histories of CVD (*p* = 0.282). Men had a significantly higher proportion in the SCHZ spectrum (61.1% vs. 42.2%, *p* = 0.000), whereas women had a higher proportion in the affective disorders spectrum (50.3% vs. 19.2%, *p* = 0.000). Men were significantly younger than women (38 ± 14 vs. 45 ± 14, *p* = 0.000) and had lower BMIs (24.8 ± 4.6 vs. 27.1 ± 5.7, *p* = 0.000) (Table 3).

**Table 3.** **Variables according to CVR and sex**.

Variable	Category	Male		Female		Chi-square	df	*p*-value
		n = 193	%	n = 173	%			
Framingham (10 yrs)	Low risk (<10)	160	82.9%	122	70.5%	7.908	1	0.005*
	More than low risk (10–100)	33	17.1%	51	29.5%			
Framingham (30 yrs)	Low risk (<10)	33	17.1%	17	9.8%	4.090	1	0.043*
	More than low risk (10–100)	160	82.9%	156	90.2%			
Cigarette use	Non-smoker	84	43.5%	128	74.0%	34.740	1	<0.001*
	Smoker	109	56.5%	45	26.0%			
BMI classification	Normal (18.5–25)	100	54.6%	59	36.0%	16.047	2	<0.001*
	Low (<18.5)	13	7.1%	7	4.3%			
	Overweight (>25)	70	38.3%	98	59.8%			
Family history - cardiovascular disease	No	180	93.3%	156	90.2%	1.158	1	0.282
	Yes	13	6.7%	17	9.8%			
Diagnosis	Schizophrenia spectrum	118	61.1%	73	42.2%	42.051	2	<0.001*
	Affective spectrum disorder	37	19.2%	87	50.3%			
	Other	38	19.7%	13	7.5%			
Education	No schooling	14	7.3%	11	6.4%	3.067	3	0.381
	Primary	43	22.3%	51	29.5%			
	High school	113	58.5%	88	50.9%			
	Technical and university	23	11.90%	23	13.30%			
History of hypertension	No	166	86.0%	150	86.7%	0.037	1	0.847
	Yes	27	14.0%	23	13.3%			
History of diabetes	No	177	91.7%	158	91.3%	0.017	1	0.896
	Yes	16	8.3%	15	8.7%			
Family history - Psychiatric illness	No	154	79.8%	127	73.4%	2.084	1	0.149
	Yes	39	20.2%	46	26.6%			
		Mean ± SD	Median (IQR)	Mean ± SD	Median (IQR)	Mann-Whitney U Test	SE	*p-*value
Age		38 ± 14	35 (27–47)	45 ± 14	45 (32–56)	4.63	1010.2	<0.001*
BMI		24.8 ± 4.6	24.2 (22.0–27.5)	27.1 ± 5.7	26.1 (23.4–30.8)	4.03	932.9	<0.001*

SE, Standard Error; BMI, Body Mass Index; IQR, Interquartile Range; SD, standard deviation; yrs, Years; CVR, cardiovascular risks. *Statistically significant.

**Fig. 1.**
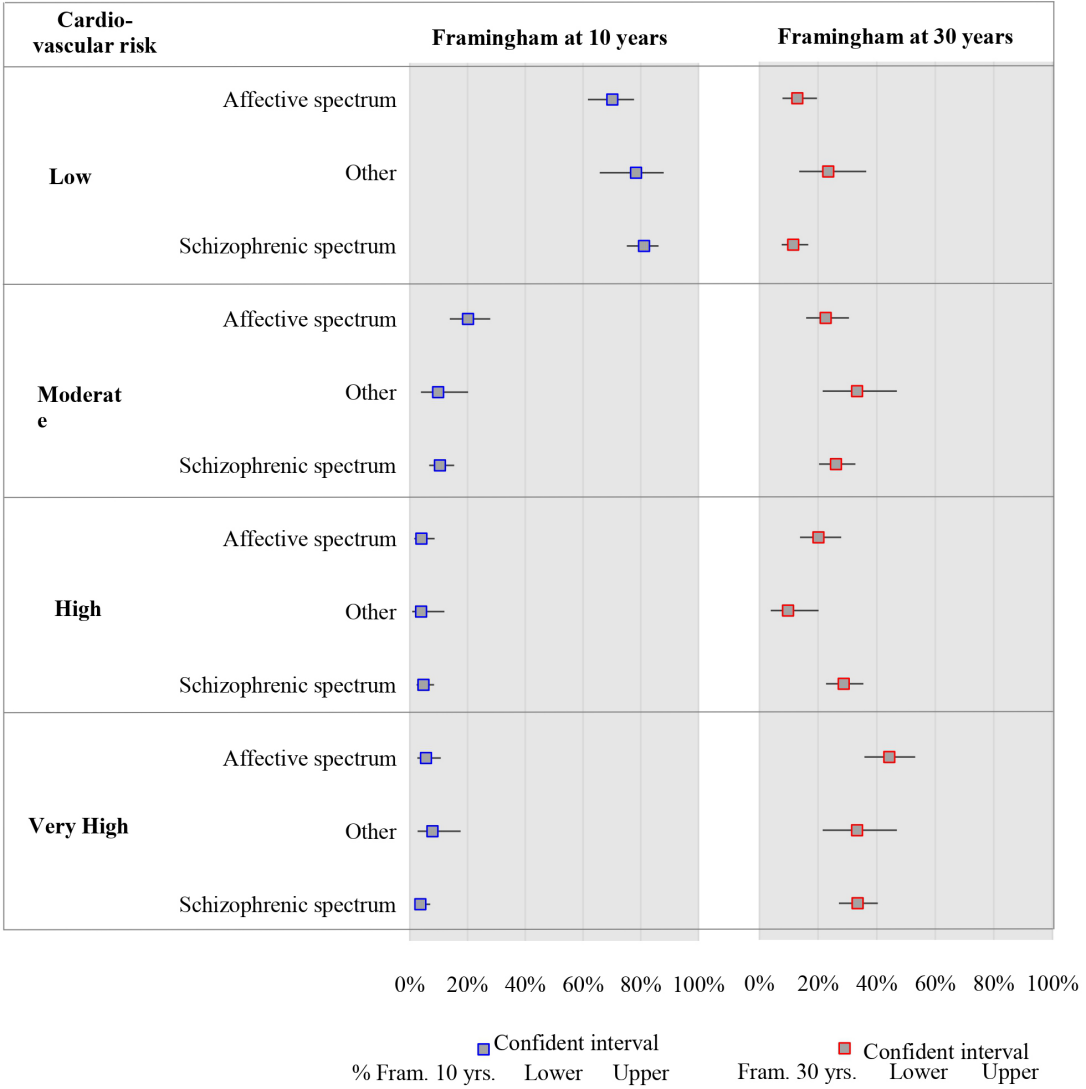
**Distribution by psychiatric diagnosis of cardiovascular risk according to Framingham at 30 and 10 years**.

Fig. 1 shows the comparison of CVR at 10 and 30 years according to the diagnosis. At 10 years, more than 60% of patients showed low CVR, while at 30 years, the percentage of high CVR increased across all diagnostic categories.

Logistic regression models showed that factors associated with the highest CVR included female sex Odds ratio (OR) = 2.01, 95% Confidence Interval (CI): (1.08–3.73), higher educational level, systolic BP >150 mmHg (OR = 7.94, 95% CI: 3.73–16.92), triglyceride >150 mg/dL (OR = 2.00, 95% CI: 1.01–3.98), fasting glucose >100 mg/dL (OR = 2.79, 95% CI: 1.40–5.39), cigarette smoking (OR = 2.22, 95% CI: 1.16–4.25), and a family history of CVD (OR = 4.05, 95% CI: 1.64–9.98) (Table 4).

**Table 4.** **Logistic regression model with OR adjusted for CVR using Framingham score at 30 years**.

Predictor		OR (95% CI)	B	*p*-value
Sex				
	Female	2.01 (1.08–3.73)	0.6981	0.028
Marital_status				
	Married	1.18 (0.53–2.66)	0.1655	0.686
Education^1^				
	Up to high school	0.25 (0.13–0.49)	–1.3863	<0.001
	Technical and university studies	0.36 (0.15–0.90)	–1.0217	0.028
Systolic BP				
	High (>140 mmHg)	7.94 (3.73–16.92)	2.0719	<0.001
Diastolic BP				
	High (>90 mmHg)	0.68 (0.31–1.48)	–0.3857	0.328
Triglycerides				
	High (>150 mg/dL)	2.00 (1.01–3.98)	0.6931	0.048
Total_cholesterol				
	High (>200 mg/dL)	1.74 (0.76–4.01)	0.5539	0.190
LDL_cholesterol				
	High (>100 mg/dL)	0.93 (0.48–1.80)	–0.0726	0.828
HDL_cholesterol				
	Low (<60 mg/dL)	1.42 (0.50–3.97)	0.3507	0.509
Fasting_glucose				
	High (>100 mg/dL)	2.79 (1.40–5.39)	1.0260	0.002
Cigarette use				
	Smoker	2.22 (1.16–4.25)	0.7975	0.016
History of cardiovascular disease				
	Family history of cardiovascular disease	4.05 (1.64–9.98)	1.3987	0.002
	Constant	0.07 (0.02–0.24)	–2.6593	<0.001

^1^Reference category: less than high school. BP, blood pressure; CI, Confidence Interval; dL, deciliters; HDL, high-density lipoprotein; LDL, low-density lipoprotein; mg, milligrams; mmHg, millimeters of mercury; OR, Odds ratio.

## Discussion

This study assessed CVR using the Framingham risk score at 10 and 30 years in a sample of patients diagnosed with SMI who required hospitalization at an institution in Colombia, South America. We identified several factors associated with increased CVR: Female sex, education below high school, systolic BP greater than 140 mmHg, high triglycerides levels (>150 mg/dL), elevated fasting glucose levels (>100 mg/dL), smoking, and a family history of CVD. Similar findings were reported in a European study, which showed that hypertension, diabetes, smoking, dyslipidemia, obesity, and metabolic syndrome contribute to higher CVR in patients with SMI compared to the general population [27]. Furthermore, the most recent state-of-the-art reviews by the American College of Cardiology Foundation and the European Society of Cardiology highlight that patients with SMI have a higher prevalence of modifiable CVR factors, such as hypertension, diabetes, and unhealthy lifestyle habits. These factors contribute to increased cardiovascular mortality in this population [6, 28].

CVDs are the leading cause of mortality and disability worldwide [10]. CVD accounts for 33% of deaths in the general population [10], while reaching 50% among patients with SMI [14, 29]. When estimating the risk of mortality from CVD using the Framingham risk score over 30 years, we found that 86.3% of patients will be at moderate, high, or very high risk with no differences by sex or diagnosis. A meta-analysis comparing patients with SMI to healthy controls using the Framingham risk score found that patients diagnosed with SCHZ have a higher CVR than those with other diagnoses. However, no differences were found when compared to healthy controls, acknowledging the heterogeneity in the studies evaluated [30]. Consistent with the rates in our study, another meta-analysis found that patients with SMI have a 53% risk of having CVD, a 78% risk of developing CVD, and an 85% risk of death from CVD [31]. Summarizing, there is substantial evidence suggesting that SMI is a significant risk factor for CVD.

We found significant differences in CVR according to sex. At both 10 and 30 years, women, unlike men, were predominantly classified as having high or very high CVR, with an odd ratio of 2.01 for these categories. These results align with those reported in a comparative study between psychiatric patients and general population in Italy [31]. The CVR at 10 years, calculated using the CUORE Project 10-year CVR algorithm, indicated that women had a higher risk than the general population while the risk for men was similar [31]. Similar findings were observed in a Danish cohort studying patients with SMI and type 2 diabetes, which reported a higher risk of CVD in women [32]. The higher risk observed in women in samples could be related to a survival bias. Since women tend to live longer than men, it is possible that the older men in our sample represent a healthier subset of their age group, which could explain why they appear healthier than the women in the sample.

The association between low education level and mortality from CVD has been described in both patients with SMI and the general population [33, 34, 35]. In our 30-year calculation, we found that patients with lower educational achievements were more likely to be at high or very high-risk of CVD, while higher education was associated with decreased CVR. This association may be due to increased access to community education, health services, medication, healthy leisure activities, and greater knowledge about healthy lifestyles.

Other modifiable risk factors have been associated with high mortality rates in patients with SMI worldwide. Patients with SMI have been reported to have up to four times higher rates of diabetes, dyslipidemia, metabolic syndrome, and hypertension [6, 19, 28, 36, 37]. Other study has found that 68% of patients with SMI smoked tobacco, 27% have hypertension, and 13% are diagnosed with diabetes [19]. In our sample, we found a tobacco consumption rate of 42.8%, hypertension by 23%, and glucose levels greater than 100 mg/dL in 20% of patients. The factors that most influenced CVR in our sample were systolic BP greater than 140 mmHg, glucose levels greater than 100 mg/dL, smoking, and triglyceride levels greater than 150 mg/dL. While there are no recent reports on the influence of these CVR factors in populations with mental illness, previous studies have shown that the combination of five specific factors—BMI, systolic BP, low-density cholesterol level, smoking, and diabetes—translates to a 10-year incidence of CVD of 57.2% in the general population [38]. Therefore, it is expected that this incidence would be higher in patients with mental illness and SMI.

Obesity is a global problem affecting 36.9% of men and 38% of women worldwide [39]. Patients with SMI have a higher risk of being overweight and developing obesity compared to individuals without mental illness [6, 18, 40], with higher rates of abdominal obesity in women [41]. In our sample, we found that almost half of participants were overweight or obese, with higher rates among women than in men. This factor becomes an important element when calculating the 30-year risk, which, as mentioned, was higher in women. It is noteworthy noting that SMI maybe be associated with unhealthy diets, drug side effects, especially atypical antipsychotics, and sedentary lifestyles, which have been described as contributing risk factors for obesity.

Elevated LDL cholesterol levels are correlated with increased mortality in the general population [10]. Patients with SMI have a significantly higher risk of hypertriglyceridemia and lower HDL levels compared to the general population [6, 18]. Genetic factors associated with hyperlipidemia have been identified in patients with SMI [18, 42, 43]. In our sample, we observed hyperlipidemia with no significant differences by sexes. These findings, in addition to other reports, highlight a potential warning sign for CVD in this population.

In this population of patients with SMI, we identified a significant risk of cardiovascular morbidity and mortality at both 10 and 30 years. Therefore, considering the most recent state-of-the-art review conducted by the European Society of Cardiology, the cardiovascular risk assessment of patients with SMI should be personalized, which involves evaluating modifiable risk factors utilizing CVR stratification tools and incorporating a multidisciplinary team to treat these patients [6].

Important limitations of this study that should be acknowledged include potential selection bias, information bias, uncertain causality, incomplete or biased data collection, and lack of control over external variables. As result, the findings should be interpreted with caution.

## Conclusions

We found a high incidence of CVD risk in the current sample of patients with SMI. Several factors were associated with an increased risk of mortality at 10 and 30 years, including patient sex, low educational levels, systolic BP greater than 140 mmHg, elevated levels of triglycerides and blood glucose, active smoking, and a family history of CVD. These findings underscore the importance of helping patients manage modifiable risk factors, such as BP, serum lipids and glucose levels, smoking habits, and potential drug side effects to positively impact survival and quality of life in this population.

In a population hospitalized for SMI in Latin America, high CVR at 10 and 30 years was identified. Factors contributing to this high CVR included demographic characteristics, comorbidities, vital signs, blood serum values, and family history of CVD. While there are non-modifiable risk factors, the presence of modifiable factors should be a point of focus, prompting early intervention to reduce the risk of premature mortality in this population in accordance with the recommendations of the European Society of Cardiology [6].

## Availability of Data and Materials

The data presented in this study are available in article.

## Supplementary Material

Supplementary material associated with this article can be found, in the online version, at https://doi.org/10.62641/aep.v53i4.1904.

## Appendix

See Table 5.

**Table 5.** **List of diagnostic codes**.

ICD-10 Code	Diagnosis	Number of patients	Diagnostic Category
F011	Multi-infarct dementia	1	Other disorders spectrum
F019	Vascular dementia, unspecified	2	Other disorders spectrum
F062	Organic delusional [schizophrenia-like] disorder	3	Other disorders spectrum
F063	Organic mood [affective] disorders	4	Other disorders spectrum
F069	Unspecified mental disorder due to brain damage and dysfunction and to physical disease	4	Other disorders spectrum
F09X	Unspecified organic or symptomatic mental disorder	2	Other disorders spectrum
F103	Mental and behavioural disorders due to use of alcohol withdrawal state	1	Other disorders spectrum
F142	Mental and behavioural disorders due to use of cocaine: dependence syndrome	1	Other disorders spectrum
F190	Mental and behavioural disorders due to multiple drug use and use of other psychoactive substances : acute intoxication	1	Other disorders spectrum
F191	Mental and behavioural disorders due to multiple drug use and use of other psychoactive substances : harmful use	1	Other disorders spectrum
F195	Mental and behavioural disorders due to multiple drug use and use of other psychoactive substances : psychotic disorder	15	Other disorders spectrum
F199	Mental and behavioural disorders due to multiple drug use and use of other psychoactive substances : unspecified mental and behavioural disorder	2	Other disorders spectrum
F200	Paranoid schizophrenia	25	SCHZ spectrum
F201	Hebephrenic schizophrenia	1	SCHZ spectrum
F202	Catatonic schizophrenia	3	SCHZ spectrum
F203	Undifferentiated schizophrenia	78	SCHZ spectrum
F208	Other schizophrenia	1	SCHZ spectrum
F209	Schizophrenia, unspecified	27	SCHZ spectrum
F220	Delusional disorder	1	SCHZ spectrum
F229	Persistent delusional disorder, unspecified	2	SCHZ spectrum
F230	Acute polymorphic psychotic disorder without symptoms of schizophrenia	1	SCHZ spectrum
F231	Acute polymorphic psychotic disorder with symptoms of schizophrenia	8	SCHZ spectrum
F232	Acute schizophrenia-like psychotic disorder	2	SCHZ spectrum
F233	Other acute predominantly delusional psychotic disorders	1	SCHZ spectrum
F239	Acute and transient psychotic disorder, unspecified	1	SCHZ spectrum
F250	Schizoaffective disorder, manic type	5	SCHZ spectrum
F251	Schizoaffective disorder, depressive type	3	SCHZ spectrum
F252	Schizoaffective disorder, mixed type	3	SCHZ spectrum
F258	Other schizoaffective disorders	2	SCHZ spectrum
F259	Schizoaffective disorder, unspecified	28	SCHZ spectrum
F302	Mania with psychotic symptoms	1	Affective disorders spectrum
F309	Manic episode, unspecified	1	Affective disorders spectrum
F311	Bipolar affective disorder, current episode manic without psychotic symptoms	1	Affective disorders spectrum
F312	Bipolar affective disorder, current episode manic with psychotic symptoms	51	Affective disorders spectrum
F313	Bipolar affective disorder, current episode mild or moderate depression	2	Affective disorders spectrum
F314	Bipolar affective disorder, current episode severe depression without psychotic symptoms	1	Affective disorders spectrum
F315	Bipolar affective disorder, current episode severe depression with psychotic symptoms	3	Affective disorders spectrum
F317	Bipolar affective disorder, currently in remission	1	Affective disorders spectrum
F318	Other bipolar affective disorders	1	Affective disorders spectrum
F319	Bipolar affective disorder, unspecified	49	Affective disorders spectrum
F323	Severe depressive episode with psychotic symptoms	6	Affective disorders spectrum
F331	Recurrent depressive disorder, current episode moderate	1	Affective disorders spectrum
F332	Recurrent depressive disorder, current episode severe without psychotic symptoms	1	Affective disorders spectrum
F333	Recurrent depressive disorder, current episode severe with psychotic symptoms	1	Affective disorders spectrum
F339	Recurrent depressive disorder, unspecified	2	Affective disorders spectrum
F412	Mixed anxiety and depressive disorder	2	Affective disorders spectrum
F429	Obsessive-compulsive disorder, unspecified	1	Affective disorders spectrum
F602	Dissocial personality disorder	1	Other disorders spectrum
F630	Pathological gambling	1	Other disorders spectrum
F711	Moderate mental retardation : significant impairment of behaviour requiring attention or treatment	4	Other disorders spectrum
F718	Moderate mental retardation : other impairments of behaviour	1	Other disorders spectrum
F719	Moderate mental retardation without mention of impairment of behaviour	1	Other disorders spectrum
F721	Moderate mental retardation without mention of impairment of behaviour	3	Other disorders spectrum
G409	Epilepsy, unspecified	1	Other disorders spectrum

ICD-10, International Statistical Classification of Diseases and Related Health Problems 10th Revision; SCHZ, Schizophrenia.
