# Supplementary material for: Cardiovascular Risk Evaluation in a Latin American Population With Severe Mental Illness: An Observational Study
Source: Actas Esp Psiquiatr. 2025 Aug 5;53(4):742–55. doi: 10.62641/aep.v53i4.1904 (PMC12353234; doi:10.62641/aep.v53i4.1904)
Supplement: Supplementary file 1 [file ActEsp-53-4-742-755-s1.docx]

**SUPPLEMENTARY MATERIAL**

**Comparative table between all variables and Framingham (10yrs)**

| Variable | Category | Framingham (10yrs) | | | | | | | | Valor p |
| --- | --- | --- | --- | --- | --- | --- | --- | --- | --- | --- |
|  |  | Low Risk (<10) | | Moderate Risk (10-20) | | High Risk (20-30) | | Very High Risk (30-100) | |  |
|  |  | n = 282 | % | n = 50 | % | n = 16 | % | n = 18 | % |  |
| Sex | Male | 160 | 56,70% | 18 | 36,00% | 6 | 37,50% | 9 | 50,00% | 0,030* |
|  | Female | 122 | 43,30% | 32 | 64,00% | 10 | 62,50% | 9 | 50,00% |  |
| Marital Status | No spouse | 247 | 87,60% | 42 | 84,00% | 11 | 68,80% | 14 | 77,80% | 0,131 |
|  | With spouse | 35 | 12,40% | 8 | 16,00% | 5 | 31,30% | 4 | 22,20% |  |
| Education | Up to Middle School | 77 | 27,30% | 26 | 52,00% | 7 | 43,80% | 9 | 50,00% | 0,003* |
|  | Up to High School | 171 | 60,60% | 16 | 32,00% | 8 | 50,00% | 6 | 33,30% |  |
|  | Technical and University Studies | 34 | 12,10% | 8 | 16,00% | 1 | 6,30% | 3 | 16,70% |  |
| Systolic BP | Low (<140 mmHg) | 238 | 84,40% | 32 | 64,00% | 6 | 37,50% | 3 | 16,70% | 0,000* |
|  | High (>140 mmHg) | 44 | 15,60% | 18 | 36,00% | 10 | 62,50% | 15 | 83,30% |  |
| Diastolic BP | Low (<90 mmHg) | 226 | 80,10% | 34 | 68,00% | 12 | 75,00% | 8 | 44,40% | 0,002* |
|  | High (>90 mmHg) | 56 | 19,90% | 16 | 32,00% | 4 | 25,00% | 10 | 55,60% |  |
| Triglycerides | Low (<150 mg/dl) | 233 | 82,60% | 37 | 74,00% | 11 | 68,80% | 12 | 66,70% | 0,132 |
|  | High (>150 mg/dl) | 49 | 17,40% | 13 | 26,00% | 5 | 31,30% | 6 | 33,30% |  |
| Total Cholesterol | Low (<200 mg/dl) | 248 | 87,90% | 42 | 84,00% | 12 | 75,00% | 9 | 50,00% | 0,000* |
|  | High (>200 mg/dl) | 34 | 12,10% | 8 | 16,00% | 4 | 25,00% | 9 | 50,00% |  |
| LDL Cholesterol | Low (<100 mg/dl) | 168 | 59,60% | 29 | 58,00% | 6 | 37,50% | 4 | 22,20% | 0,007* |
|  | High (>100 mg/dl) | 114 | 40,40% | 21 | 42,00% | 10 | 62,50% | 14 | 77,80% |  |
| HDL Cholesterol | High (>60 mg/dl) | 29 | 10,30% | 4 | 8,00% | 1 | 6,30% | 2 | 11,10% | 0,915 |
|  | Low (<60 mg/dl) | 253 | 89,70% | 46 | 92,00% | 15 | 93,80% | 16 | 88,90% |  |
| Fasting Glucose | Low (<100 mg/dl) | 241 | 85,50% | 34 | 68,00% | 11 | 68,80% | 8 | 44,40% | 0,000* |
|  | High (>100 mg/dl) | 41 | 14,50% | 16 | 32,00% | 5 | 31,30% | 10 | 55,60% |  |
| Cigarette Use | Non-Smoker | 166 | 58,90% | 32 | 64,00% | 8 | 50,00% | 6 | 33,30% | 0,071 |
|  | Former Smoker | 11 | 3,90% | 3 | 6,00% | 2 | 12,50% | 1 | 5,60% |  |
|  | Light Smoker (<10 years) | 66 | 23,40% | 4 | 8,00% | 3 | 18,80% | 4 | 22,20% |  |
|  | Moderate Smoker (10-20 years) | 25 | 8,90% | 8 | 16,00% | 1 | 6,30% | 4 | 22,20% |  |
|  | Heavy Smoker (>20 years) | 14 | 5,00% | 3 | 6,00% | 2 | 12,50% | 3 | 16,70% |  |
| BMI classification | Low (<18.5) | 17 | 6,30% | 1 | 2,10% | 1 | 7,10% | 1 | 5,90% | 0,207 |
|  | Normal (18.5-25) | 131 | 48,90% | 15 | 31,30% | 6 | 42,90% | 7 | 41,20% |  |
|  | Overweight (25-30) | 66 | 24,60% | 20 | 41,70% | 6 | 42,90% | 4 | 23,50% |  |
|  | Obese (>30) | 54 | 20,10% | 12 | 25,00% | 1 | 7,10% | 5 | 29,40% |  |
| Antipsychotic Medication | Amisulpride | 8 | 2,80% | 1 | 2,00% | 0 | 0,00% | 0 | 0,00% | 0,008* |
|  | Aripiprazole | 5 | 1,80% | 2 | 4,00% | 1 | 6,30% | 1 | 5,60% |  |
|  | Clozapine | 61 | 21,60% | 8 | 16,00% | 1 | 6,30% | 1 | 5,60% |  |
|  | Haloperidol | 8 | 2,80% | 2 | 4,00% | 0 | 0,00% | 0 | 0,00% |  |
|  | Olanzapine | 59 | 20,90% | 4 | 8,00% | 3 | 18,80% | 2 | 11,10% |  |
|  | Paliperidone | 4 | 1,40% | 1 | 2,00% | 0 | 0,00% | 2 | 11,10% |  |
|  | Quetiapine | 48 | 17,00% | 17 | 34,00% | 7 | 43,80% | 9 | 50,00% |  |
|  | Risperidone | 89 | 31,60% | 15 | 30,00% | 4 | 25,00% | 3 | 16,70% |  |
| History of cardiovascular disease | No family history of cardiovascular disease | 266 | 94,30% | 40 | 80,00% | 14 | 87,50% | 16 | 88,90% | 0,007* |
|  | Family history of cardiovascular disease | 16 | 5,70% | 10 | 20,00% | 2 | 12,50% | 2 | 11,10% |  |
| Family Psychiatric History | None | 213 | 75,50% | 41 | 82,00% | 12 | 75,00% | 15 | 83,30% | 0,569 |
|  | Other | 30 | 10,60% | 4 | 8,00% | 2 | 12,50% | 0 | 0,00% |  |
|  | Schizophrenia | 17 | 6,00% | 5 | 10,00% | 1 | 6,30% | 1 | 5,60% |  |
|  | Bipolar | 13 | 4,60% | 0 | 0,00% | 1 | 6,30% | 2 | 11,10% |  |
|  | Anxiety/Depression | 9 | 3,20% | 0 | 0,00% | 0 | 0,00% | 0 | 0,00% |  |
| Dx | Schizophrenia | 155 | 55,00% | 20 | 40,00% | 9 | 56,30% | 7 | 38,90% | 0,188 |
|  | Affective | 87 | 30,90% | 25 | 50,00% | 5 | 31,30% | 7 | 38,90% |  |
|  | Other | 40 | 14,20% | 5 | 10,00% | 2 | 12,50% | 4 | 22,20% |  |

**Comparative table between all variables and Framingham (30yrs)**

| Variable | Category | Framingham (30yrs) | | | | | | | | | Valor p | |  |
| --- | --- | --- | --- | --- | --- | --- | --- | --- | --- | --- | --- | --- | --- |
|  |  | Low Risk (<10) | | Moderate Risk (10-20) | | High Risk (20-30) | | Very High Risk (30-100) | | |  | |  |
|  |  | n = 50 | % | n = 95 | % | n = 85 | % | | n = 136 | % | |  | |
| Sex | Male | 33 | 66,0% | 59 | 62,1% | 40 | 47,1% | | 61 | 44,9% | | 0,010* | |
|  | Female | 17 | 34,0% | 36 | 37,9% | 45 | 52,9% | | 75 | 55,1% | |  |  |
| Marital Status | No spouse | 49 | 98,0% | 78 | 82,1% | 77 | 90,6% | | 110 | 80,9% | | 0,009* | |
|  | With spouse | 1 | 2,0% | 17 | 17,9% | 8 | 9,4% | | 26 | 19,1% | |  |  |
| Education | Up to Middle School | 16 | 32,0% | 19 | 20,0% | 23 | 27,1% | | 61 | 44,9% | | 0,001* | |
|  | Up to High School | 31 | 62,0% | 66 | 69,5% | 47 | 55,3% | | 57 | 41,9% | |  |  |
|  | Technical and University Studies | 3 | 6,0% | 10 | 10,5% | 15 | 17,6% | | 18 | 13,2% | |  |  |
| Systolic BP | Low (<140 mmHg) | 47 | 94,0% | 82 | 86,3% | 69 | 81,2% | | 81 | 59,6% | | 0,000* | |
|  | High (>140 mmHg) | 3 | 6,0% | 13 | 13,7% | 16 | 18,8% | | 55 | 40,4% | |  |  |
| Diastolic BP | Low (<90 mmHg) | 48 | 96,0% | 77 | 81,1% | 65 | 76,5% | | 90 | 66,2% | | 0,000* | |
|  | High (>90 mmHg) | 2 | 4,0% | 18 | 18,9% | 20 | 23,5% | | 46 | 33,8% | |  |  |
| Triglycerides | Low (<150 mg/dl) | 47 | 94,0% | 79 | 83,2% | 66 | 77,6% | | 101 | 74,3% | | 0,020* | |
|  | High (>150 mg/dl) | 3 | 6,0% | 16 | 16,8% | 19 | 22,4% | | 35 | 25,7% | |  |  |
| Total Cholesterol | Low (<200 mg/dl) | 48 | 96,0% | 85 | 89,5% | 71 | 83,5% | | 107 | 78,7% | | 0,014* | |
|  | High (>200 mg/dl) | 2 | 4,0% | 10 | 10,5% | 14 | 16,5% | | 29 | 21,3% | |  |  |
| LDL Cholesterol | Low (<100 mg/dl) | 35 | 70,0% | 60 | 63,2% | 42 | 49,4% | | 70 | 51,5% | | 0,036* | |
|  | High (>100 mg/dl) | 15 | 30,0% | 35 | 36,8% | 43 | 50,6% | | 66 | 48,5% | |  |  |
| HDL Cholesterol | High (>60 mg/dl) | 6 | 12,0% | 7 | 7,4% | 11 | 12,9% | | 12 | 8,8% | | 0,573 | |
|  | Low (<60 mg/dl) | 44 | 88,0% | 88 | 92,6% | 74 | 87,1% | | 124 | 91,2% | |  |  |
| Fasting Glucose | Low (<100 mg/dl) | 46 | 92,0% | 86 | 90,5% | 70 | 82,4% | | 92 | 67,6% | | 0,000* | |
|  | High (>100 mg/dl) | 4 | 8,0% | 9 | 9,5% | 15 | 17,6% | | 44 | 32,4% | |  |  |
| Cigarette Use | Non-Smoker | 38 | 76,0% | 47 | 49,5% | 50 | 58,8% | | 77 | 56,6% | | 0,000* | |
|  | Former Smoker | 1 | 2,0% | 3 | 3,2% | 4 | 4,7% | | 9 | 6,6% | |  |  |
|  | Light Smoker (<10 years) | 11 | 22,0% | 32 | 33,7% | 18 | 21,2% | | 16 | 11,8% | |  |  |
|  | Moderate Smoker (10-20 years) | 0 | 0,0% | 11 | 11,6% | 9 | 10,6% | | 18 | 13,2% | |  |  |
|  | Heavy Smoker (>20 years) | 0 | 0,0% | 2 | 2,1% | 4 | 4,7% | | 16 | 11,8% | |  |  |
| BMI classification | Low (<18.5) | 6 | 13,0% | 4 | 4,4% | 4 | 4,9% | | 6 | 4,6% | | 0,009* | |
|  | Normal (18.5-25) | 29 | 63,0% | 49 | 54,4% | 32 | 39,5% | | 49 | 37,7% | |  |  |
|  | Overweight (25-30) | 6 | 13,0% | 21 | 23,3% | 25 | 30,9% | | 44 | 33,8% | |  |  |
|  | Obese (>30) | 5 | 10,9% | 16 | 17,8% | 20 | 24,7% | | 31 | 23,8% | |  |  |
| Antipsychotic Medication | Amisulpride | 1 | 2,0% | 4 | 4,2% | 1 | 1,2% | | 3 | 2,2% | | 0,232 | |
|  | Aripiprazole | 1 | 2,0% | 0 | 0,0% | 4 | 4,7% | | 4 | 2,9% | |  |  |
|  | Clozapine | 8 | 16,0% | 23 | 24,2% | 20 | 23,5% | | 20 | 14,7% | |  |  |
|  | Haloperidol | 3 | 6,0% | 1 | 1,1% | 3 | 3,5% | | 3 | 2,2% | |  |  |
|  | Olanzapine | 12 | 24,0% | 20 | 21,1% | 16 | 18,8% | | 20 | 14,7% | |  |  |
|  | Paliperidone | 0 | 0,0% | 2 | 2,1% | 2 | 2,4% | | 3 | 2,2% | |  |  |
|  | Quetiapine | 10 | 20,0% | 13 | 13,7% | 16 | 18,8% | | 42 | 30,9% | |  |  |
|  | Risperidone | 15 | 30,0% | 32 | 33,7% | 23 | 27,1% | | 41 | 30,1% | |  |  |
| History of cardiovascular disease | No family history of cardiovascular disease | 47 | 94,0% | 91 | 95,8% | 80 | 94,1% | | 118 | 86,8% | | 0,057 | |
|  | Family history of cardiovascular disease | 3 | 6,0% | 4 | 4,2% | 5 | 5,9% | | 18 | 13,2% | |  |  |
| Family Psychiatric History | None | 40 | 80,0% | 73 | 76,8% | 65 | 76,5% | | 103 | 75,7% | | 0,463 | |
|  | Other | 5 | 10,0% | 9 | 9,5% | 10 | 11,8% | | 12 | 8,8% | |  |  |
|  | Schizophrenia | 3 | 6,0% | 4 | 4,2% | 6 | 7,1% | | 11 | 8,1% | |  |  |
|  | Bipolar | 2 | 4,0% | 4 | 4,2% | 1 | 1,2% | | 9 | 6,6% | |  |  |
|  | Anxiety/Depression | 0 | 0,0% | 5 | 5,3% | 3 | 3,5% | | 1 | 0,7% | |  |  |
| Dx | Schizophrenia | 22 | 44,0% | 50 | 52,6% | 55 | 64,7% | | 64 | 47,1% | | 0,016* | |
|  | Affective | 16 | 32,0% | 28 | 29,5% | 25 | 29,4% | | 55 | 40,4% | |  |  |
|  | Other | 12 | 24,0% | 17 | 17,9% | 5 | 5,9% | | 17 | 12,5% | |  |  |

**Sociodemographic variables of the Framingham risk score at 10 and 30 years.**

| Variable | Category | Low Risk | | Moderate Risk | | High Risk | | Very High Risk | | P value |
| --- | --- | --- | --- | --- | --- | --- | --- | --- | --- | --- |
|  |  | (<10) | | (10-20) | | (20-30) | | (30-100) | |  |
| Framingham (10years) | | n = 282 | % | n = 50 | % | n = 16 | % | n = 18 | % |  |
| Diagnosis | Schizophrenia spectrum | 155 | 55.0 | 20 | 40.0 | 9 | 56.3 | 7 | 38.9 | 0.188 |
|  | Affective disorder spectrum | 87 | 30.9 | 25 | 50.0 | 5 | 31.3 | 7 | 38.9 |  |
|  | Other disorder spectrum | 40 | 14.2 | 5 | 10.0 | 2 | 12.5 | 4 | 22.2 |  |
| Sex | Male | 160 | 56.7 | 18 | 36.0 | 6 | 37.5 | 9 | 50.0 | 0.030* |
|  | Female | 122 | 43.3 | 32 | 64.0 | 10 | 62.5 | 9 | 50.0 |  |
| Marital Status | Single | 247 | 87.6 | 42 | 84.0 | 11 | 68.8 | 14 | 77.8 | 0.131 |
|  | Married | 35 | 12.4 | 8 | 16.0 | 5 | 31.3 | 4 | 22.2 |  |
| Education | No schooling | 18 | 6.4 | 5 | 10.0 | 1 | 6.3 | 1 | 5.6 | 0.012* |
|  | Primary | 59 | 20.9 | 21 | 42.0 | 6 | 37.5 | 8 | 44.4 |  |
|  | High School | 171 | 60.6 | 16 | 32.0 | 8 | 50.0 | 6 | 33.3 |  |
|  | Technical and University | 34 | 12.1 | 8 | 16.0 | 1 | 6.3 | 3 | 16.7 |  |
| Framingham (30years) | | n = 50 | % | n = 95 | % | n = 85 | % | n = 136 | % | P value |
| Diagnosis | Schizophrenia spectrum | 22 | 44.0 | 50 | 52.6 | 55 | 64.7 | 64 | 47.1 | 0.016* |
|  | Affective disorder spectrum | 16 | 32.0 | 28 | 29.5 | 25 | 29.4 | 55 | 40.4 |  |
|  | Other disorder spectrum | 12 | 24.0 | 17 | 17.9 | 5 | 5.9 | 17 | 12.5 |  |
| Sex | Male | 33 | 66.0 | 59 | 62.1 | 40 | 47.1 | 61 | 44.9 | 0.010* |
|  | Female | 17 | 34.0 | 36 | 37.9 | 45 | 52.9 | 75 | 55.1 |  |
| Marital Status | Single | 49 | 98.0 | 78 | 82.1 | 77 | 90.6 | 110 | 80.9 | 0.009* |
|  | Married | 1 | 2.0 | 17 | 17.9 | 8 | 9.4 | 26 | 19.1 |  |
| Education | No schooling | 3 | 6.0 | 4 | 4.2 | 8 | 9.4 | 10 | 7.4 | 0.001* |
|  | Primary | 13 | 26.0 | 15 | 15.8 | 15 | 17.6 | 51 | 37.5 |  |
|  | High School | 31 | 62.0 | 66 | 69.5 | 47 | 55.3 | 57 | 41.9 |  |
|  | Technical and University | 3 | 6.0 | 10 | 10.5 | 15 | 17.6 | 18 | 13.2 |  |
